# Actions of Quercetin, a Polyphenol, on Blood Pressure

**DOI:** 10.3390/molecules22020209

**Published:** 2017-01-29

**Authors:** Yoshinori Marunaka, Rie Marunaka, Hongxin Sun, Toshiro Yamamoto, Narisato Kanamura, Toshio Inui, Akiyuki Taruno

**Affiliations:** 1 Department of Molecular Cell Physiology, Kyoto Prefectural University of Medicine, Kyoto 602-8566, Japan; marurie@koto.kpu-m.ac.jp (R.M.); crystal@koto.kpu-m.ac.jp (H.S.); inui-cl@nike.eonet.ne.jp (T.I.); taruno@koto.kpu-m.ac.jp (A.T.); 2 Department of Bio-Ionomics, Kyoto Prefectural University of Medicine, Kyoto 602-8566, Japan; 3 Japan Institute for Food Education and Health, St. Agnes’ University, Kyoto 602-8013, Japan; 4 Department of Dental Medicine, Kyoto Prefectural University of Medicine, Kyoto 602-8566, Japan; yamamoto@koto.kpu-m.ac.jp (T.Y.); kanamura@koto.kpu-m.ac.jp (N.K.); 5 Saisei Mirai Clinics, Moriguchi 570-0012, Japan

**Keywords:** flavonoid, quercetin, Na^+^-K^+^-2Cl^−^ cotransporter 1, epithelial Na^+^ channel, cytosolic Cl^−^ concentration

## Abstract

Disorder of blood pressure control causes serious diseases in the cardiovascular system. This review focuses on the anti-hypertensive action of quercetin, a flavonoid, which is one of the polyphenols characterized as the compounds containing large multiples of phenol structural units, by varying the values of various blood pressure regulatory factors, such as vascular compliance, peripheral vascular resistance, and total blood volume via anti-inflammatory and anti-oxidant actions. In addition to the anti-inflammatory and anti-oxidant actions of quercetin, we especially describe a novel mechanism of quercetin’s action on the cytosolic Cl^−^ concentration ([Cl^−^]_c_) and novel roles of the cytosolic Cl^−^ i.e., (1) quercetin elevates [Cl^−^]_c_ by activating Na^+^-K^+^-2Cl^−^ cotransporter 1 (NKCC1) in renal epithelial cells contributing to Na^+^ reabsorption via the epithelial Na^+^ channel (ENaC); (2) the quercetin-induced elevation of [Cl^−^]_c_ in renal epithelial cells diminishes expression of ENaC leading to a decrease in renal Na^+^ reabsorption; and (3) this reduction of ENaC-mediated Na^+^ reabsorption in renal epithelial cells drops volume-dependent elevated blood pressure. In this review, we introduce novel, unique mechanisms of quercetin’s anti-hypertensive action via activation of NKCC1 in detail.

## 1. Introduction

Blood pressure is regulated by many factors such as: (1) cardiac output, vascular compliance (reciprocal of elastance), and peripheral vascular resistance; (2) total blood volume dependent on body fluid volume; (3) nervous control systems (autonomic sympathetic and parasympathetic nervous systems, baroreflex and baroreceptors); and (4) renin-angiotensin system-regulating vascular resistance and renin-angiotensin-aldosterone system-regulating blood volume. Polyphenols containing large multiples of phenol structural units are widely distributed in plants, such as vegetables, soybeans, and fruits, and are well known to provide us with health benefits including anti-hypertensive actions via anti-inflammatory and anti-oxidant actions. Polyphenols show anti-inflammatory actions by inhibiting activity and/or production of inflammation-inducing enzymes such as cyclooxygenase [1,2,3], lipoxygenase [3,4] and TNF-α [1,5,6,7,8], etc. Polyphenols also have anti-oxidant abilities via interaction with anti-oxidant enzymes, such as heme oxygenase-1 (HO-1) [9], increases in the activities of various antioxidant enzymes, such as catalase and glutathione peroxidase [10], radical scavenging ability [10,11], and redox regulation by binding to β-actin, a cytoskeletal protein, and Keap1, a scaffold protein bound to the actin cytoskeleton controlling cytoprotective enzyme genes through sulfhydryl modification [12]. Thus, polyphenols protect vascular cells, including endothelial and smooth muscle cells, from inflammation and oxidative stress via these anti-inflammatory and anti-oxidant actions, and maintain normal vascular compliance and resistance, providing their anti-hypertensive actions.

**Figure 1:**
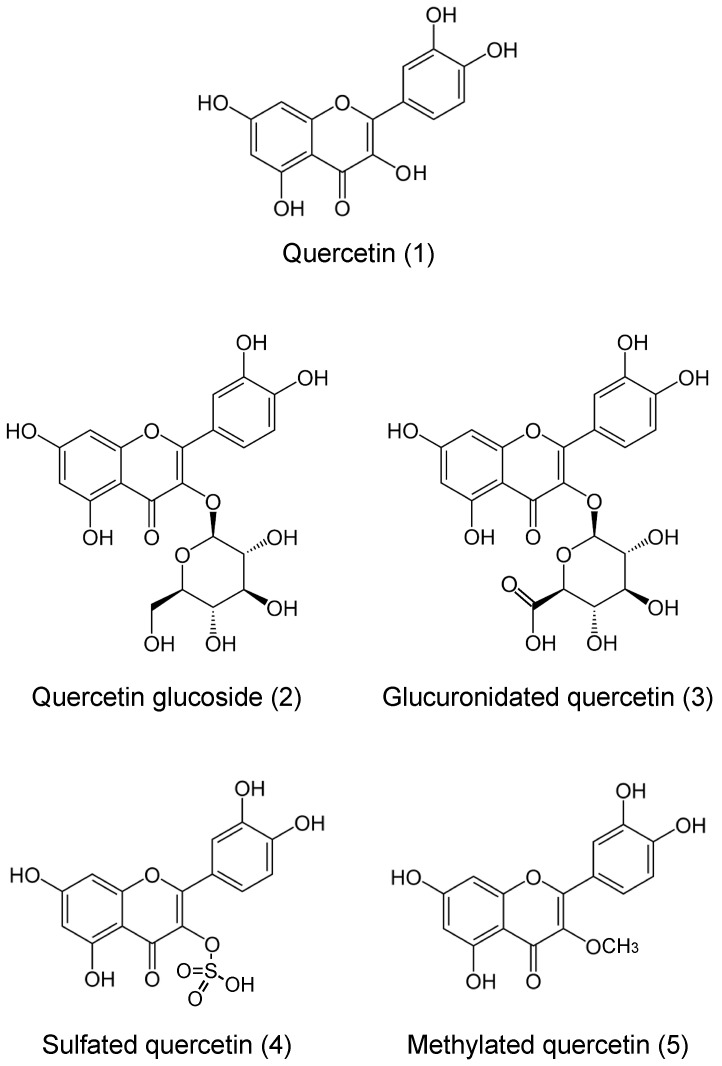
Structures of quercetin (1) and its metabolites (2–5).

In addition to these actions, growing evidence has demonstrated that polyphenols regulate ion transporters and channels [13,14]. It is generally accepted that ion environments regulated by ion transporters and channels are essentially important to maintain homeostasis of various cellular and body functions: e.g., blood pressure dependent on body fluid volume via regulation of epithelial Na^+^ channels (ENaC) [15,16], and hyperglycemia in insulin-resistant diabetes mellitus caused by low interstitial pH [17,18,19,20,21]. Quercetin activates Na^+^-K^+^-2Cl^−^ cotransporter 1 (NKCC1), leading to elevation of the cytosolic Cl^−^ concentration ([Cl^−^]_c_) [22], which down-regulates gene expression of ENaC [23]. As a result of the aforementioned actions, polyphenols show various beneficial effects on body functions including regulation of blood pressure via diminution of vascular contraction [24] and renal Na^+^ reabsorption by influencing ENaC gene expression [15,23,25] and Na^+^,K^+^-ATPase activity [26]. This review will focus on the anti-hypertensive actions of quercetin (Figure 1) enriched in onion skins and ingested as a part of common diets [27], mainly via regulation of total blood volume dependent on body fluid volume, and also slightly through regulation of vascular compliance (reciprocal of elastance) and resistance. 

## 2. How Do We Take Up and Absorb Flavonoids into the Inside of the Body?

Suzuki and Hara [28] have thoroughly summarized how humans absorb daily flavonoids, including quercetin. Therefore, in this review, we provide information on quercetin actions while avoiding information given by Suzuki and Hara [28]. Quercetin exists as such (aglycone) and in glycosylated forms (Figure 1). Quercetin aglycone is lipophilic, based on its structure (Figure 1). Due to this lipophilic property quercetin aglycone is easily absorbed through the phospholipid bilayer of intestinal mucosal epithelial cells [28]. On the other hand, the glycosylated form of quercetin is non-lipophilic and commonly found in plants, such as fruits, vegetables, and soybeans. This glycosylated form of quercetin is not easily absorbed into the body due to its non-lipophilic property. However, Day et al. have reported that lactase phlorizin hydrolase (LPH) expressed in the brush border membrane of the rat intestine hydrolyzes the glycosylated form of quercetin to the aglycone form [29]. Whereas quercetin aglycone is absorbed through the phospholipid bilayers of intestinal mucosal epithelial cells by diffusion, the monoglucoside form of quercetin is also absorbed into the cytosol of intestinal cells by sodium glucose transporter-1 (SGLT-1) expressed on the brush border membrane of rat intestine [30] and the apical membrane of human small intestinal model Caco-2 cells [31]. This monoglucoside-type of quercetin taken up into the cells is reported to be deglycosylated by β-glucosidase located in the cytosolic space of human intestinal cells [32]. Thus, quercetin molecules are taken up into the intestinal cells by simple diffusion through the phospholipid bilayer and by facilitated diffusion mediated via SGLT-1, and exist as aglycone in the cytosolic space in both cases of humans and rats. Then, in the cells the quercetin aglycone is glucuronidated, sulfated, or methylated (Figure 1) and these modified types of quercetin molecules enter circulation [33]. The major metabolites in rats are di- and tri-conjugates of quercetin, such as glucuronidated, sulfate and/or methylated ones [33,34], while in humans, quercetin-3′-*O*-glucuronide is the major circulating metabolite [34]. These observations suggest that significant species differences occur in major circulating metabolites of quercetin. Therefore, although rat is one of the good models, we should consider these specific differences in metabolites of quercetin in circulation to study the quercetin actions on body and cell functions. 

## 3. Actions of Quercetin on Blood Pressure in Human and Model Animals

In humans, oral intake of quercetin of 150~730 mg/day between four and ten weeks shows anti-hypertensive actions. A randomized, double-blind, placebo-controlled, crossover study indicates that 730 mg/day quercetin intake for four weeks decreases systolic and diastolic blood pressures in stage 1 hypertensive patients, but has no effects on systolic or diastolic blood pressure with prehypertension [35]. A study in human with metabolic syndrome [36] has reported that 150 mg/day quercetin intake for five weeks decreases systolic blood pressure. Further, a double-blind randomized clinical trial [37] carried out on 72 women with type 2 diabetes indicates that 500 mg/day quercetin intake for 10 weeks significantly decreases systolic blood pressure, although diastolic pressure is not significantly affected by the quercetin intake. Serban et al. [38] summarize several human meta-analysis of randomized controlled trials, indicating that quercetin intake >500 mg/day over eight weeks, but not ≤500 mg/day, significantly decreases systolic and diastolic blood pressures.

In model animals, oral administration of quercetin also shows an anti-hypertensive action [15,16]. Choi et al. [39] have reported direct actions of quercetin on vascular tension of the aorta obtained from renal hypertensive rats. In this ex vivo study [39], they have demonstrated that quercetin (10^−5^ M) acutely potentiates vascular relaxation induced by acetylcholine in 2K1C hypertensive rats, suggesting that quercetin shows its anti-hypertensive action by diminishing elastance of blood vessels. Galisteo et al. [40] have reported the action of chronic oral intake of quercetin on systolic blood pressure and vascular function in deoxycorticosterone acetate (DOCA)-salt hypertensive rats. Quercetin enhances endothelial-dependent aortic dilatations in rats developed with DOCA-salt-induced hypertension in a similar manner to verapamil, a Ca^2+^ channel blocker, which reduces blood pressure [40]. However, this study [40] indicates that quercetin is more effective in improving hypertension than verapamil in volume-expanded hypertension. This clearly indicates that, in addition to vascular relaxation, a decrease in body fluid volume is partly to account for the hypertensive action of quercetin. This action of quercetin could be mainly applied to reduce the elevated systolic blood pressure with age caused by a decrease in compliance of the aorta, although quercetin is also effective on volume-expanded hypertension. Further, quercetin shows an anti-hypertensive action in spontaneously hypertensive rats [41]. An in vivo study [42] in spontaneously hypertensive rats reports that quercetin increases the sensitivity of the parasympathetic component of the baroreflex, decreasing the mean artery pressure. Further, quercetin has been reported to attenuate the noradrenaline-induced contraction in a perfused mesenteric resistance vascular bed isolated from spontaneously hypertensive rats [43]. Quercetin also interrupts the renin-angiotensin system via diminution of mRNA expression of angiotensin-converting enzyme (ACE) and inhibition of ACE activity [44]. A study [45] performed in NaCl-induced volume-expanded hypertensive rats indicates that quercetin intake of 50, 100, and 150 mg/kg/day decreases systolic and diastolic pressures in a dose-dependent manner, supporting that quercetin has an anti-hypertensive action in volume-expanded hypertension. Thus, quercetin shows its anti-hypertensive actions via regulation of vascular compliance (the reciprocal of elastance) and resistance, total blood volume dependent on body fluid volume, autonomic nervous system, and the renin-angiotensin system.

## 4. Anti-Inflammatory and Anti-Oxidant Actions of Quercetin and Its Influence on Blood Pressure

Quercetin has been reported to have an anti-inflammatory action in both human and model animals by inhibiting activities of cyclooxygenase [1,2,3], lipoxygenase [3,4], expression of cyclooxygenase and production of PGE_2_ [2], diminishing production of interleukin (IL) -1α [7], PKC-dependent IgE-mediated proinflammatory mediator, IL-6 and IL-8 [8], TNF-α [1,5,6,7,8], and poly (ADP-ribose) polymerase-1 (PARP-1) [46], down-regulating vascular cell adhesion molecule-1 (VCAM-1) and cluster differentiation (CD) 80 [47], etc. Quercetin also shows anti-oxidant actions via radical scavenging ability [48] and by interacting with anti-oxidant enzymes, such as heme oxygenase-1 (HO-1), which protects oxidative stress H_2_O_2_-induced apoptosis, and reduces intracellular ROS production and mitochondria dysfunction [9]. Thus, quercetin shows its anti-hypertensive actions [35,49] via possible mechanisms dependent on the anti-inflammatory and anti-oxidant actions, such as protection of cardiovascular cells associated with a decrease in triacylglycerol concentration and an increase in HDL-cholesterol concentration [50], endothelium-dependent vasodilation due to an increased NO production [51], and prevention of endothelial cell apoptosis [52], etc. Due to the anti-hypertensive effect of quercetin via vascular resistance has already been extensively discussed in many review articles [53,54,55,56,57]. In the following sections we will focus on mechanisms of the anti-hypertensive action of quercetin via control of body fluid volume by altering the intracellular ion environments.

## 5. Roles of ENaC in Control of Blood Pressure

**Figure 2:**
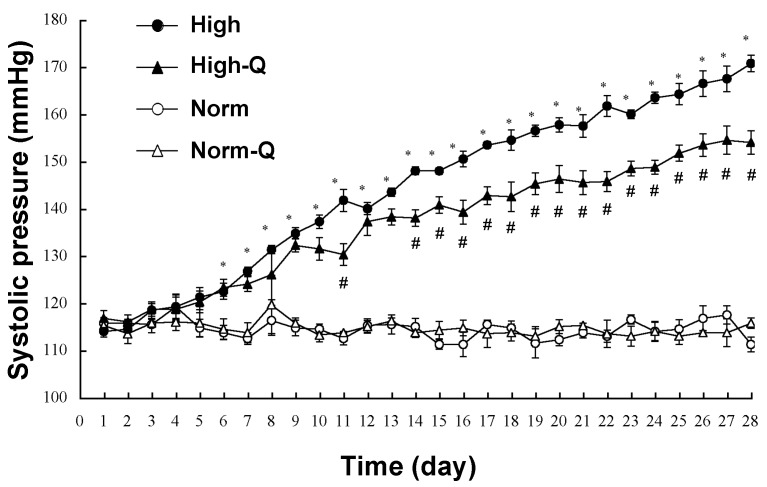
Systolic arterial pressure of Dahl salt-sensitive hypertensive rats. The systolic arterial pressure is represented as means ± SEM (*n* = 4 in each group). Norm: normal diet; Norm-Q: normal diet with quercetin; High: high-NaCl diet; and High-Q: high-NaCl diet with quercetin. * Significant difference between high-NaCl diet (High) and normal diet (Norm) at the same experimental time (*p* < 0.05). # Significant difference between high-NaCl with quercetin (High-Q) and high-NaCl diet (High) at the same experimental time (*p* < 0.05). Quercetin (10 mg/kg/day) was orally applied. The systolic blood pressure, which was measured under an awaking condition by the tail cuff method. Adopted from [15] with allowance for non-profit use of the figure.

**Figure 3:**
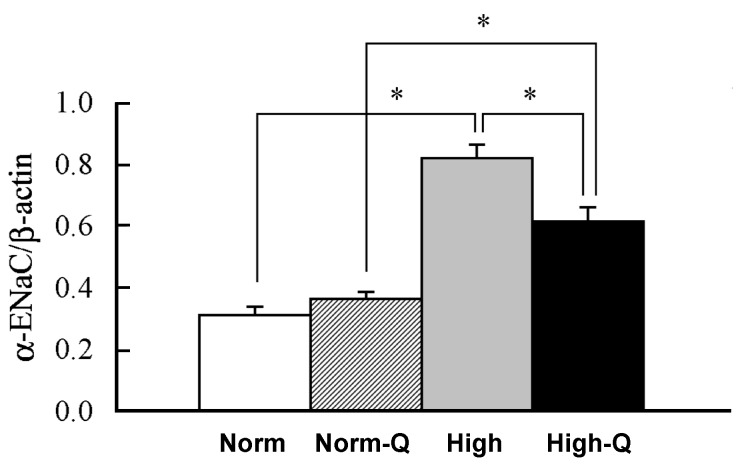
mRNA expression of αENaC in the kidney of Dahl salt-sensitive hypertension rats. The amount of mRNA expression of αENaC is expressed as the ratio to β-actin expression. Data are represented as means ± SEM obtained from four rats. Norm: normal diet; Norm-Q: normal diet with quercetin; High: high-NaCl diet and High-Q: high-NaCl diet with quercetin. * Significant difference at the level of *p* < 0.05. Quercetin (10 mg/kg/day) was orally applied. Adopted from [15] with allowance for non-profit use of the figure.

Salt-sensitive hypertension is caused by an expansion of body fluid volume via an increase of water absorption due to high salt intake-induced elevation of body fluid osmolarity. Dahl salt-sensitive hypertensive rats are commonly used to study the molecular mechanism of salt-sensitive hypertension. Quercetin action on salt-sensitive hypertension has been studied in this animal model. High salt intake causes hypertension in Dahl salt-sensitive hypertensive rats (Figure 2) [15]. Daily oral intake of 10 mg/kg of quercetin significantly suppresses the high salt intake-induced elevation of the systolic blood pressure in Dahl salt-sensitive hypertensive rats (Figure 2), and shows a tendency to increase urine volume and NaCl excretion into urine with no effects on the plasma aldosterone level [15,58,59]. This oral administration of quercetin diminishes mRNA expression of the pore-forming α subunit of ENaC (Figure 3) [15], essentially regulating the amount of Na^+^ reabsorption in the kidney, which is a key regulator of blood pressure via determination of body fluid volume [16,60]. Namely, the activity of individual ENaC, and the number of ENaC expressed at the apical membrane of renal collecting ducts, critically control blood pressure [61,62,63].

Canessa et al., in Rossier’s research group, have cloned ENaC subunits from rat colon [64,65]: in 1993, they cloned the pore-forming α subunit of ENaC and in 1994 they cloned two other essential subunits, establishing that ENaC consists of three subunits, α, β, and γ [65]. Later, the δ subunit was also discovered in humans [66]. Patients with Liddle’s syndrome, an autosomal dominant form of hereditary hypertension, carry gain-of-function mutations in ENaC genes, which disrupt ubiquitination sites of the channel. The deficiency of ubiquitination causes a constitutively increased number of ENaCs located in the apical membrane of the collecting duct epithelial cell of the kidney [67,68,69,70,71,72,73,74,75,76], leading to constitutively high ENaC-mediated renal Na^+^ reabsorption and, thereby elevated body fluid volume. The phenomenon observed in Liddle’s syndrome strongly indicates that the expression of ENaCs plays an essential role in control of blood pressure. In Dahl salt-sensitive hypertensive rats, high salt intake elevates blood pressure associated with an increase in αENaC expression (Figure 3) abnormally responding to aldosterone [15], the plasma level of which is significantly lowered by high salt intake via the renin-angiotensin-aldosterone system [15]. This suggests that the increase in ENaC expression induced by high salt intake would elevate blood pressure due to the expansion of blood volume resulting from the elevation of body fluid volume caused by an increase in renal Na^+^ reabsorption even with a low plasma aldosterone level due to high salt intake. This means that high salt-sensitive hypertension would be due to disorders in the regulation of ENaC expression by aldosterone [58]. Orally administered quercetin of 10 mg/kg/day significantly diminishes the elevated αENaC expression under a low aldosterone condition caused by high salt intake in Dahl salt-sensitive hypertensive rats, while no effects are seen in control rats (Figure 3) [15]. In an in vitro experiment using a renal epithelial cell line, the quercetin action on ENaC expression has been studied [23], indicating that quercetin diminishes ENaC gene expression. This indicates that quercetin would be a useful compound showing an anti-hypertensive action on high salt-sensitive hypertension without any significant influence on the basal blood pressure in normotensive individuals with normal salt intake, although the quercetin action on ENaC expression should be further examined in various systems.

## 6. Anti-Hypertensive Action of Quercetin via Down-Regulation of ENaC Expression Dependent on Elevation of [Cl^−^]_c_ Due to Its Stimulatory Action on NKCC-1

**Figure 4:**
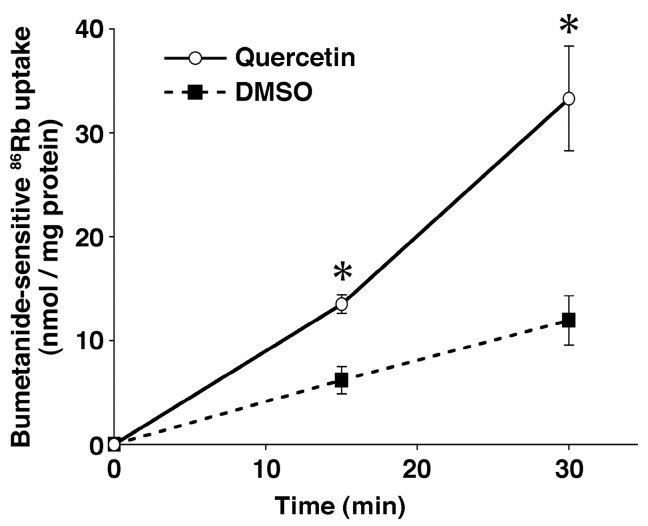
Effect of quercetin (10 μM) on NKCC1 activity in rat pheochromocytoma PC12 cells. NKCC1 activity was estimated by measuring the bumetanide-sensitive ^86^Rb uptake. Data are represented as means ± SEM. * *p* < 0.01 vs. DMSO. *n* = 3 for t = 0 min, *n* = 5 for t = 15 min, *n* = 7 for t = 30 min. This observation indicates that quercetin stimulates activity of NKCC1. Adopted from [22] with allowance for non-profit use of the figure.

In this section, we introduce the quercetin action on ENaC expression via stimulation of NKCC1, which regulates transepithelial Cl^−^ secretion and [Cl^−^]_c_ by participating in Cl^−^ uptake in epithelial cells [77]. Quercetin has been reported to stimulate NKCC1 (Figure 4) expressed in neural cells, which mediates uptake of Cl^−^ into the cytosolic space driven by the Na^+^, K^+^-ATPase-generated electro-chemical potential of Na^+^ [22,78,79,80]. The stimulatory action of quercetin on NKCC1 has been also reported in renal epithelial cells [14,25,81]. Although cAMP is indicated to act as a second messenger of quercetin action [82] and quercetin stimulates translocation of an unknown factor elevating NKCC1 activity to the basolateral membrane expressing NKCC1 [80], we still have little information on how quercetin stimulates NKCC1 at the molecular level. One possible molecular pathway of the quercetin action is mediated through sulfhydryl modification, as mentioned above [12]. Nevertheless, it is notable that quercetin modifies cellular functions via the elevation of [Cl^−^]_c_ by stimulating NKCC1 [22], whereas many studies indicate that quercetin influences various cellular and body functions via its anti-inflammatory and anti-oxidant actions, as mentioned above. 

Interestingly, it has been reported that reduction of [Cl^−^]_c_ up-regulates ENaC expression [23], while elevation of [Cl^−^]_c_ down-regulates ENaC expression [23]. Quercetin elevates [Cl^−^]_c_ by stimulating NKCC1 (Figure 4) [14,22], indicating that the quercetin-induced down-regulation of ENaC expression (Figure 3) would be mediated through elevation of [Cl^−^]_c_ [23]. This is supported by an observation that expression of ENaC is also down-regulated by a Cl^−^ channel blocker, 5-nitro-2-(3-phenylpropylamino)benzoic acid (NPPB), which increases [Cl^−^]_c_ by blocking Cl^−^ release from the cytosolic space via Cl^−^ channels [23]. The quercetin-induced reduction of ENaC expression diminishes the amount of ENaC-mediated Na^+^ reabsorption in epithelial cells of renal collecting ducts and lowers high salt intake-induced high blood pressure by compensating (reducing) elevated body fluid volume [15,23]. Although this unique action of quercetin on ENaC expression is interesting, we should further investigate the molecular mechanism of the quercetin action on ENaC expression in various systems. The investigation on the quercetin regulation of ENaC expression via control of [Cl^−^]_c_ mediated through NKCC1 would lead us to an interesting, novel therapy against hypertension.

## 7. Conclusions

**Figure 5:**
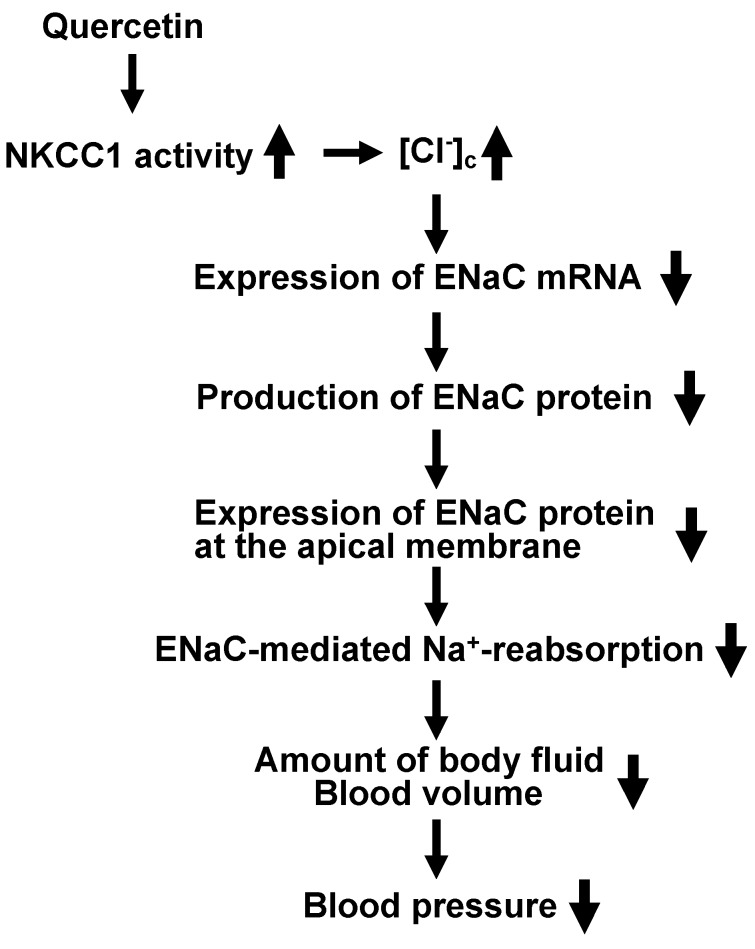
A possible mechanism of quercetin actions on blood pressure via modification of extracellular fluid volume mediated by ENaC-mediated Na^+^ reabsorption in the kidney.

Quercetin shows its anti-hypertensive actions via modification of various factors controlling blood pressure, such as vascular compliance (reciprocal of elastance) and resistance, total blood volume dependent on body fluid volume, autonomic nervous system, and renin-angiotensin-system via anti-inflammatory and anti-oxidative abilities. In addition to these abilities, quercetin has a unique action on regulation of ENaC gene expression. This action is mediated via control of [Cl^−^]_c_ by activating NKCC1. In this review, we show the novel idea on the role of “cytosolic Cl^−^“ in the anti-hypertensive action of quercetin on volume-dependent regulation of blood pressure. This novel idea regarding the anti-hypertensive action of quercetin via NKCC1 stimulation on ENaC-mediated Na^+^ reabsorption in the kidney is summarized in Figure 5.

## Abbreviations

The following abbreviations are used in this manuscript:

ACE	angiotensin-converting enzyme
CD	cluster differentiation
[Cl^−^]_c_	cytosolic Cl^−^ concentration
DOCA	deoxycorticosterone acetate
ENaC	epithelial Na^+^ channel
HO-1	heme oxygenase-1
IL	interleukin
LPH	lactase phlorizin hydrolase
NKCC1	Na^+^-K^+^-2Cl^−^ cotransporter 1
NPPB	(5-nitro-2-(3-phenylpropylamino)benzoic acid
PARP-1	poly (ADP-ribose) polymerase-1
SGLT-1	sodium glucose transporter-1
VCAM-1	vascular cell adhesion molecule-1
